# Gender-Specific Patterns in Nonsyndromic Cleft Lip and/or Palate: A Four-Year Retrospective Study From Telangana, India

**DOI:** 10.7759/cureus.106744

**Published:** 2026-04-09

**Authors:** Praveen Kumar M, Kumar Satish Ravi, Srinivas Reddy Gosla, Vasavi Mohan, Praveen Kumar Neela, Nainika Krishnan, Fatema Ali Khan

**Affiliations:** 1 Anatomy, National Institute of Medical Sciences and Research, NIMS University Rajasthan, Jaipur, IND; 2 Oral and Maxillofacial Surgery, GSR Institute of Craniomaxillofacial and Facial Plastic Surgery, Hyderabad, IND; 3 Genetics, Department of Genetics and Molecular Medicine, Vasavi Medical and Research Centre, Hyderabad, IND; 4 Orthodontics and Dentofacial Orthopaedics, Kamineni Institute of Dental Sciences, Narketpally, IND

**Keywords:** cleft lip, cleft palate, epidemiology, gender, prevalence

## Abstract

Cleft lip and/or palate (CL/P) is a common congenital condition with known gender differences, but region-specific data from Telangana are limited. This hospital-based retrospective cross-sectional study evaluated 665 (n=665) patients treated between 2020 and 2024 to analyze gender distribution across cleft types. Clefts were categorized as cleft lip (CL), cleft palate (CP), and cleft lip with palate (CLP), and statistical analysis included the chi-square test, odds ratio (OR), and Z-test. CLP was the most common presentation (n=393, 59.10%), followed by CL (n=234, 35.19%) and CP (n=38, 5.71%). A clear gender pattern was observed, with males more frequently affected in CLP (n=233, 35.03%) and CL (n=126, 18.95%), while CP was more common in females (n=23, 3.45%). The association between gender and cleft type was statistically significant (χ² = 6.37, df = 2, p = 0.041). Males were more likely to present with CLP compared to CP (OR = 2.23; 95% CI: 1.13-4.41), which was further supported by the Z-test (p = 0.018). Overall, a significant gender disparity was observed in cleft distribution in Telangana, highlighting the need for gender-sensitive screening and management strategies.

## Introduction

Cleft lip and/or palate (CL/P) cases are among the most common congenital anomalies globally, with notable geographic, ethnic, and gender-based variations. The incidence of CL/P is estimated to be around one in 700 live births worldwide [1]. In India, studies have documented variable prevalence patterns, highlighting the role of genetic and environmental interactions [2]. A systematic review conducted in Mexico highlighted environmental risk factors such as pollution exposure and the role of folic acid pathways in cleft prevention [3]. Additionally, a study from the population-based Registry of Congenital Defects of Tuscany found an association between orofacial clefts and maternal risk factors [4].

Gender disparity in CL/P has been widely reported. Cleft lip (CL; with or without palate) generally shows a male predilection, while isolated cleft palate (CP) tends to be more frequent in females [5,6]. However, gender-specific prevalence data are limited in regional populations such as Telangana, India. This study presents a detailed analysis of gender differences in cleft type distribution from a large clinical dataset.

The aim of this study was to categorize cleft cases by type and gender, analyze the association between cleft type and gender, and compare local gender distribution based on a large clinical dataset.

## Materials and methods

Ethical approval for this study was obtained from the Institutional Ethics Committee of Nims University Rajasthan, Jaipur (Approval No.: IEC/P-505/2024, dated January 12, 2024) and the Institutional Ethics Committee of GSR Institute of Craniofacial and Plastic Surgery, Hyderabad (Approval No.: GSR ICFS/2024/001-N, dated January 17, 2024). The study was conducted in accordance with the Declaration of Helsinki, and patient confidentiality and data anonymity were strictly maintained.

This retrospective study was conducted at the GSR Institute of Craniofacial and Plastic Surgery (GSRICES), a high-volume tertiary care center in Hyderabad, Telangana, India. The institute specializes in diagnosing and treating craniofacial anomalies, including CL/P. This study reviewed data collected over four years, from September 2020 to August 2024. From a pool of approximately 4000 records, 665 eligible patient records were selected using a simple random sampling method, ensuring unbiased selection. As this study was a hospital-based retrospective study including all eligible cases during the study period, no formal sample size calculation was performed. These patients were registered and treated at GSRICES for cleft-related conditions during the specified period.

Inclusion and exclusion criteria

Inclusion criteria included patients with a confirmed clinical diagnosis of CL, CP, or cleft lip and palate (CLP). Only nonsyndromic cases were included. Syndromic cases (such as Pierre Robin sequence and Stickler syndrome), records with incomplete data, and patients with unrelated craniofacial anomalies were excluded from the study. The study was conducted using anonymized data, and no direct patient interaction was involved.

Data collection procedure

Patient data were retrieved from hospital medical records and entered into a structured Microsoft Excel (Redmond, WA, USA) spreadsheet for analysis. The variables collected included gender (male/female) and cleft type (CL, CP, CLP).

Statistical analysis

The collected data were analyzed using IBM SPSS Statistics for Windows, version 26.0 (IBM Corp., Armonk, NY, USA). Descriptive statistics were used to compute frequencies and percentages for categorical variables (cleft type and gender). To determine the relationship between cleft type and gender, the chi-square test of independence was used. An odds ratio (OR) with a 95% confidence interval (CI) was calculated to compare the odds of male involvement in CLP versus CP. A Z-test was also performed to compare proportions between groups. A p-value of <0.05 was considered statistically significant.

## Results

A total of 665 patients with cleft anomalies were included in the study, comprising 374 males (56.24%) and 291 females (43.76%), resulting in an overall male-to-female ratio (M:F) of 1.29:1. The mean age of the patients was six years, with ages ranging from infancy to adulthood.

The distribution of cleft types is presented in Table 1. CLP was the most common type (n=393, 59.10%), followed by CL (n=234, 35.19%), while isolated CP (n=38, 5.71%) was the least frequent.

**Table 1:** Distribution of cleft types by gender percentages based on total (n=665) CL – Cleft Lip; CP – Cleft Palate; CLP – Cleft Lip and Palate; M:F – Male-to-Female ratio

Cleft Type	Male	Female	Total	% Male	% Female	Total (%)	Ratio (M:F)
CL	126	108	234	18.95%	16.24%	35.19%	1.17:1
CP	15	23	38	2.25%	3.45%	5.71%	0.65:1
CLP	233	160	393	35.03%	24.06%	59.10%	1.46:1
Total	374	291	665	56.24%	43.76%	100%	1.29:1

A clear gender-based pattern was observed across cleft types (Table 1). Both CLP (n=233 males, 35.03%) and CL (n=126 males, 18.95%) showed male predominance, whereas CP demonstrated a higher occurrence in females (n=23, 3.45%).

The association between gender and cleft type was found to be statistically significant (χ² = 6.37, df = 2, p = 0.041).

Further analysis using OR, calculated based on the cross-tabulation of gender and cleft types (CLP vs. CP) as shown in Table 2, showed that males were more likely to present with CLP compared to CP (OR = 2.23; 95% CI: 1.13-4.41; p = 0.041). The Z-test corroborated this finding, revealing a significant disparity in the male proportion between the CLP and CP groups (Z = 2.36, p = 0.018).

**Table 2:** Cross-tabulation of gender and cleft type (CLP vs CP) with odds ratio The odds ratio (OR) for males presenting with CLP compared to CP was 2.23 (95% CI: 1.13–4.41; p = 0.041). CLP – Cleft Lip and Palate; CP – Cleft Palate

Cleft Type	Male (n)	Female (n)	Total (n)
CLP	233	160	393
CP	15	23	38

Overall, these findings indicate a statistically significant association between gender and the distribution of cleft types in the study population.

## Discussion

CL/P is a common congenital condition with varied clinical presentations and a complex multifactorial etiology. Gender-based differences in the occurrence and type of clefts have been consistently reported, reflecting both biological and sociocultural influences. In this study, conducted at the GSR Institute of Craniofacial and Plastic Surgery, 665 cases were analyzed, revealing a significant association between cleft type and gender. These findings provide insight into how gender distribution patterns of cleft types present in this region and how they compare with other populations. All percentages reported in this study are based on the total sample size (n=665).

In the present study, CLP was the most common cleft type (n=393, 59.10%), followed by CL (n=234, 35.19%) and CP (n=38, 5.71%). A significant male predominance was observed in CLP (M:F 1.46:1) and CL (M:F 1.17:1), while CP alone showed a distinct female predominance (M:F 0.65:1). A similar pattern has been reported in earlier studies where M:F ratios for CLP typically range from 1.5 to 2:1, which may be related to the greater vulnerability of primary palate fusion in males.

Our findings differ from those reported in a study conducted in Maharashtra by Shivlani et al. (2023) [6], which found a higher prevalence of orofacial cleft in males (n=1600, 53.83%) than in females (46.17%). In India, similar gender-based patterns have been reported in various regional studies. A landmark study by Reddy SG et al. (2010) [2] in Andhra Pradesh documented a clear male predominance; 65% of the children born were males with clefts, echoing our findings. In a population-based study in Chandigarh by Singh et al. (2022) [7], researchers also observed more cleft cases in males (53.4%). A study conducted by Gupta et al. (2024) [8] in Eastern India reported a relatively lower male predominance (M:F ~1.1:1) compared to the present study (1.46:1), suggesting a milder gender difference in their population.

A 12-year retrospective study by N. Farshidfar et al. (2023) [9] in Southern Iran showed distinct results: CP (40.75%), CLP (48.32%), and CL (10.93%), although a significant male predominance was observed across all cleft types (P ≤ 0.001). Similarly, Martelli et al. (2007) [10] in Brazil reported nearly comparable frequencies of CLP (39.68%), CL (38.81%), and CP (22.23%). They also observed that CLP was more common in males, who were about 2.5 times more affected than females.

These gender-specific patterns may be influenced by embryological and hormonal differences. The fusion of the primary palate (involved in CL) occurs earlier in embryogenesis. This stage may be relatively more susceptible in males. In contrast, the secondary palate (involved in CP) fuses later, and female fetuses are thought to be more vulnerable to anomalies at this stage [11].

A review by Vyas et al. (2020) [5] highlighted consistent male predominance in CL and CLP across Indian regions, with CP more common in females. These findings further support the biological basis of gender differences in cleft distribution and reinforce the credibility of our results within the Indian context. However, there are also regional variations. A study by Jamilian et al. (2017) [12] from Tehran reported a slight male predominance in cleft cases. The study also identified several associated risk factors, including consanguinity, family history, and maternal nutritional factors. However, these factors were not specifically linked to differences in gender distribution.

Further comparison with additional recent studies strengthens the interpretation of the present findings. A global review by Mossey and Modell (2012) [1] reported a male predominance in CLP of approximately 1.5:1, which closely aligns with the present study (1.46:1). Similarly, Diwana et al. (2021) [13] from North India observed comparable trends with male predominance in CLP, supporting consistency within the Indian context.

Beyond these findings, international comparisons further highlight variability. Studies from Korea (Kim et al., 2002) [14] have reported a more pronounced male predominance in CL (M:F ≈ 2.1:1) and CLP (M:F ≈ 2.5:1), along with a female predominance in CP. In contrast, the present study shows a similar overall pattern but with a less marked male predominance (CL: 1.17:1; CLP: 1.46:1). This suggests that while the general trend in gender distribution remains consistent, the extent of these differences may vary across populations.

While biological factors may explain part of the gender disparity, sociocultural influences also play an important role. In India, female children are often less likely to receive timely and adequate healthcare compared to males [15]. Similarly, a study from Bihar under the Rashtriya Bal Swasthya Karyakram (RBSK) [16] reported a male predominance in cleft cases (M:F ≈ 3:2), suggesting that differences in healthcare access and reporting may influence the observed distribution. In rural settings, delayed care and underreporting among female children may further contribute to this pattern [17].

Our findings reveal a significant gender disparity in cleft deformities, with males showing a higher likelihood of developing CLP compared to CP alone, as indicated by an odds ratio of 2.23 (95% CI: 1.13-4.41). The chi-square analysis confirmed a significant association between gender and cleft type in this cohort. Since this study was conducted in a tertiary referral center, the findings may partly reflect referral patterns rather than the true population distribution. Therefore, the results should be interpreted with caution when generalizing to the wider population.

These findings are important from a public health perspective. The preponderance of CLP and CL in males and the higher frequency of isolated CP in females underscore the importance of improved neonatal screening and referral services. Gender-sensitive health awareness programs are especially needed in communities with poor access to healthcare [18].

Limitations

This study has certain limitations that should be considered while interpreting the findings. As a single-center, hospital-based study, the results may not fully represent the broader population, as referral patterns can influence the type and severity of cases seen. The retrospective design depends on existing medical records, which may be subject to incomplete or inconsistent documentation. Additionally, as the study included only nonsyndromic cleft cases, the findings cannot be generalized to syndromic clefts, which may show different epidemiological patterns. Furthermore, potential influencing factors such as socioeconomic status, environmental exposures, and maternal risk factors were not assessed.

Future directions

Future research should focus on conducting prospective, community-based studies to minimize referral bias and better represent underserved populations. Additionally, exploring sociodemographic variables such as maternal health, education, and folic acid intake may help identify modifiable risk factors. Establishing a state-level cleft registry could further improve understanding of incidence and outcomes. Collaborative efforts between surgical centers and public health institutions are also essential to enhance early detection and access to care.

## Conclusions

In this study, a clear pattern was observed in the distribution of CL/P, with males more commonly affected by CL and CLP, while CP was seen more frequently in females. The association between gender and cleft type was also found to be statistically significant. These findings provide valuable insight into gender-based variations in cleft distribution and may help support early diagnosis and more effective patient care. However, studies involving larger and more diverse populations are needed to further explore the underlying factors contributing to these differences.

## References

[1] Mossey PA, Modell B (2012). Epidemiology of oral clefts 2012: an international perspective. Front Oral Biol.

[2] Reddy SG, Reddy RR, Bronkhorst EM, Prasad R, Ettema AM, Sailer HF, Bergé SJ (2010). Incidence of cleft Llip and palate in the state of Andhra Pradesh, South India. Indian J Plast Surg.

[3] López-Verdín S, Solorzano-López JA, Bologna-Molina R, Molina-Frechero N, Tremillo-Maldonado O, Toral-Rizo VH, González-González R (2024). The frequency of risk factors for cleft lip and palate in Mexico: a systematic review. Diagnostics (Basel).

[4] Santoro M, Mezzasalma L, Coi A, Pierini A (2024). Orofacial clefts and maternal risk factors: a population-based case-control study. Children (Basel).

[5] Vyas T, Gupta P, Kumar S, Gupta R, Gupta T, Singh HP (2020). Cleft of lip and palate: a review. J Family Med Prim Care.

[6] Shivlani VI, Niranjane PP, Diagavane PS, Madhu PP (2023). Demographic profile of patients with cleft lip and palate anomaly: 15-year experience from a tertiary care hospital and teaching institute in Wardha district of Maharashtra, India. Int J Clin Pediatr Dent.

[7] Singh S, Singh D, Utreja A (2022). Epidemiology of cleft lip and palate among infants born in Chandigarh. J Postgrad Med Educ Res.

[8] Gupta AR, Ahluwalia R, Chugh T, Jha MS, Natarajan PV (2024). Clinical, demographic, and socioeconomic profile of cleft lip and palate in eastern India. J Pharm Bioallied Sci.

[9] Farshidfar N, Ajami S, Sahmeddini S, Goli A, Foroutan HR (2023). Epidemiological and spatiotemporal descriptive analysis of patients with nonsyndromic cleft lip and/or palate: a 12-year retrospective study in southern Iran. Biomed Res Int.

[10] Martelli-Junior H, Porto LV, Martelli DR, Bonan PR, Freitas AB, Della Coletta R (2007). Prevalence of nonsyndromic oral clefts in a reference hospital in the state of Minas Gerais, Brazil, between 2000-2005. Braz Oral Res.

[11] Bernheim N, Georges M, Malevez C, De Mey A, Mansbach A (2006). Embryology and epidemiology of cleft lip and palate. B-ENT.

[12] Jamilian A, Sarkarat F, Jafari M, Neshandar M, Amini E, Khosravi S, Ghassemi A (2017). Family history and risk factors for cleft lip and palate patients and their associated anomalies. Stomatologija.

[13] Diwana VK, Sharma R, Gupta S (2019). Clinical and epidemiological profile of patients with cleft lip and palate anomaly: 10-year experience from a tertiary care center in the sub-Himalayan state of Himachal Pradesh in Northern India. J Nat Sci Biol Med.

[14] Kim S, Kim WJ, Oh C, Kim JC (2002). Cleft lip and palate incidence among the live births in the Republic of Korea. J Korean Med Sci.

[15] Willis JR, Kumar V, Mohanty S (2009). Gender differences in perception and care-seeking for illness of newborns in rural Uttar Pradesh, India. J Health Popul Nutr.

[16] Datta S, Singh VK (2025). Epidemiological pattern of cleft lip and palate under Rashtriya Bal Swasthya Karyakram project in the state of Bihar: a cross-sectional hospital-based study. J Family Med Prim Care.

[17] Chung KY, Sorouri K, Wang L, Suryavanshi T, Fisher D (2019). The impact of social stigma for children with cleft lip and/or palate in low-resource areas: a systematic review. Plast Reconstr Surg Glob Open.

[18] Beaty TH, Marazita ML, Leslie EJ (2016). Genetic factors influencing risk to orofacial clefts: today's challenges and tomorrow's opportunities. F1000Res.

